# Colloidal Oatmeal in the Management of Atopic Dermatitis and Related Xerotic Dermatoses: A Case Series

**DOI:** 10.7759/cureus.117651

**Published:** 2026-10-08

**Authors:** B. S. Chandrashekar, Abir Saraswat, Kiran Godse, Ganesh Pai, Aseem Sharma, Nikhil Bangale, Harshad Malve, Someshwar Rayasam

**Affiliations:** 1 Department of Dermatology, Cutis Hospital, Bengaluru, IND; 2 Department of Dermatology, Indushree Skin Clinic, Lucknow, IND; 3 Department of Dermatology, D.Y. Patil University, School of Medicine, Mumbai, IND; 4 Department of Dermatology, Derma Care, Mangalore, IND; 5 Department of Dermatology, Skin Saga, Mumbai, IND; 6 Department of Medical and Safety Sciences, JNTL Consumer Health (India) Pvt. Ltd (Kenvue), Mumbai, IND

**Keywords:** atopic, colloidal oatmeal, dermatitis, eczema, pruritus, quality of life

## Abstract

Introduction: Atopic dermatitis (AD) is the most common chronic inflammatory skin disorder, manifesting as recurrent, itchy eczema that often fluctuates with seasons. Since skin barrier impairment is an initiating event in AD, emollients form the foundational therapy in its management. Clinical evidence supports the use of colloidal oatmeal-based emollients as both primary and adjunctive therapy; however, literature from individual case studies remains limited, particularly in India.

Methods: In this retrospective multicenter case series, medical records of patients aged ≥12 years with AD and associated dermatological conditions who received a colloidal oatmeal-based moisturizer (Aveeno^®^ Dermexa Cream; Kenvue, JNTL Consumer Health [India] Pvt. Ltd., Mumbai, India) between 2024 and 2025 were reviewed. Demographic, clinical, treatment, and outcome data, including symptom improvement, disease severity, physician-assessed response, and flare-free intervals, were collected from medical records.

Results: A total of six patients were included over a one-year period. Of these, four had AD, one had severe eczema craquelé, and one had lichen amyloidosis with xerosis, all of whom were managed with a colloidal oatmeal-based moisturizer. Clinical observations showed improvements in skin hydration, erythema, scaling, and patient comfort, with remission maintained in some cases. These findings suggest a potential supportive role for colloidal oatmeal-based moisturizer in acute and maintenance management, although treatment efficacy and corticosteroid-sparing effects cannot be established from this case series.

Conclusion: Colloidal oatmeal-based moisturizer improved symptoms and skin hydration in patients with AD and related dermatological conditions. These findings support its role in remission maintenance and long-term disease management.

## Introduction

Atopic dermatitis (AD), a prevalent type of eczema, represents the most common chronic inflammatory skin disorder. The pathogenesis of AD involves genetic predisposition and environmental triggers, resulting in epidermal and immune abnormalities [1]. Globally, AD affects 204.05 million individuals, with an estimated overall prevalence of 2.6%. This includes 101.27 million adults and 102.78 million children, with corresponding prevalence rates of 2.0% and 4.0%, respectively [2]. In India, the prevalence of AD in children ranges from 3.1% to 7.21%, while estimates for the general population, including all age groups, vary between 0.98% and 9.2% [3].

Clinically, AD manifests as recurrent, itchy eczema that fluctuates with seasons. Patients often present with comorbid allergic conditions, including asthma, rhinoconjunctivitis, food allergies, and immediate hypersensitivity reactions [4]. The onset of AD, often associated with pruritus, frequently occurs during infancy and is characterized by dry skin, eczematous lesions, and potential lichenification [1]. AD and chronic eczema have been described as chronic xerotic skin diseases [5]. In the present case series, patients with AD and other conditions characterized by prominent xerotic features are collectively described as having related xerotic dermatoses. Dermatological conditions, such as lichen amyloidosis and xerosis, also share features of chronic pruritus and epidermal barrier dysfunction, contributing to the overall disease burden. Lichen amyloidosis has been associated with AD, potentially via interleukin (IL)-31-mediated inflammation and itching, which may contribute to AD in predisposed individuals, with a reported prevalence of approximately 0.8% [6]. Xerosis is another frequent and burdensome manifestation in patients with AD [7]. Persistent pruritus, recurrent flares, clinical symptoms, and associated comorbidities significantly impair the quality of life (QoL) of both patients and their families, often affecting sleep, daily functioning, and psychosocial well-being [8].

The management of AD includes avoiding triggers, daily skin care, anti-inflammatory treatments, and supportive therapies. Daily skin care involves the application of emollients twice daily, which is crucial for preventing skin dryness [1]. Since skin barrier impairment is an initiating event in AD pathogenesis, emollients serve as the foundational therapy in the management of AD [9]. Despite the proven efficacy of conventional anti-inflammatory treatments for AD, long-term management with topical corticosteroids remains challenging due to concerns regarding side effects and poor adherence [10,11].

In 2003, the Food and Drug Administration approved colloidal oatmeal as a safe and effective Category I skin protectant [12]. Rich in barrier-supporting beta-glucans and starches, antioxidant avenanthramides, hydrating proteins and lipids, and saponins that aid gentle cleansing, colloidal oatmeal targets key features of eczema and AD, including skin barrier dysfunction, oxidative stress, reduced hydration, and inflammation, thereby supporting skin barrier function, improving hydration, and soothing sensitive skin while maintaining a favorable safety profile [9]. It also promotes lactic acid production, a component of the natural moisturizing factor, further improving skin hydration [9].

Clinical evidence supports the use of colloidal oatmeal-based emollients as both primary [13-15] and adjunctive [16] treatment in the management of AD. Although these international studies have highlighted their clinical benefits, published clinical observations from Indian patients treated with colloidal oatmeal-based moisturizers remain limited, particularly in routine clinical practice and in patients with AD and related xerotic dermatoses. Considering potential variations in skin type, environmental exposures, and disease expression, the present case series, which includes Indian patients treated with colloidal oatmeal-based moisturizers, may provide additional real-world clinical observations. The present retrospective case series presents patients (age ≥12 years) with AD and related xerotic dermatoses, including severe eczema craquelé and lichen amyloidosis with xerosis, from multiple centers between 2024 and 2025. The objective of this retrospective case series was to describe clinical outcomes, including symptom improvement, and explore the potential supportive role of a colloidal oatmeal-based moisturizer in patients with AD and related xerotic dermatoses, including severe eczema craquelé and lichen amyloidosis with xerosis, without seeking to establish treatment efficacy or causality.

## Materials and methods

In this case series, the medical records of patients aged ≥12 years with AD and associated dermatological conditions, including eczema craquelé and lichen amyloidosis with xerosis, managed at multiple dermatology centers between January 2024 and January 2025 were retrospectively reviewed. Relevant cases were identified and selected randomly by the treating dermatologists from patients presenting to their outpatient departments during the study period. The participating centers included Shree Skin Centre, Mumbai; Cutis Hospital, Bangalore; Indushree Skin Clinic, Lucknow; Skin Saga, Mumbai; and Derma Care, Mangalore. Patients who received a colloidal oatmeal-based moisturizer (Aveeno® Dermexa Cream; Kenvue, JNTL Consumer Health (India) Pvt. Ltd., Mumbai, India) as part of their routine clinical management were included. The moisturizer contained water, glycerin, panthenol, distearyldimonium chloride, petrolatum, isopropyl palmitate, cetyl alcohol, dimethicone, Avena sativa (oat) kernel flour, Avena sativa kernel oil, steareth-20, benzalkonium chloride, ceramide NP, Avena sativa kernel extract, sodium chloride, potassium sorbate, benzyl alcohol, and tocopheryl acetate. The moisturizer was typically recommended for topical application twice daily. The duration of treatment was individualized according to clinical requirements and was at the discretion of the treating dermatologist. Concomitant therapies were not standardized across cases and were used according to individual clinical requirements; details of concomitant treatments, where available, are described for each case.

Data on demographic characteristics, clinical presentation, treatment details, follow-up findings, and clinical outcomes were extracted from medical records. Clinical outcomes included changes in symptoms such as pruritus and xerosis, clinical observations of disease status, physician-assessed clinical response, and flare-free intervals where available. Clinical outcomes were assessed based on the clinical observations of the treating dermatologist at each participating center. Photographic documentation of the affected areas before and after treatment was done for the cases presented. Follow-up duration and timing of clinical assessments were individualized according to clinical requirements and patient concerns and were not based on a fixed schedule across cases. Tolerability and adverse events, if any, were recorded based on clinical observations. As no intervention was performed specifically for research purposes and no identifiable patient information was disclosed, formal ethics committee approval was not required. All data were anonymized prior to analysis, and written informed consent for publication of clinical details and de-identified photographs was obtained from adult patients and from the parents or legal guardians of patients aged <18 years, as applicable.

## Results

Six patients were identified who fulfilled the inclusion criteria. Of the six patients, four were diagnosed with AD, one with severe eczema craquelé, and one with lichen amyloidosis with xerosis that were managed by the colloidal oatmeal-based moisturizer.

Case 1 (AD)

A 19-year-old man presented with intermittent itchy lesions over the neck and skin folds that had been present since childhood. The patient reported a recent flare-up involving the neck, underarms, and forearms over the past week. Seasonal changes typically triggered flare-ups. The patient had a medical history of asthma and was undergoing treatment. The medical history also included intermittent use of topical steroids for skin lesions. There was no family history of AD. On examination, diffuse erythema, scaling, and excoriation marks were observed over the neck, bilateral axillae, and cubital fossae. Mild scaling and erythema were noted on the forehead. Diffuse xerosis was present across the skin. Examination of the integuments and general examination revealed no additional abnormalities. The patient was diagnosed with AD.

As initial treatment, the patient was advised to use a non-soap cleanser for bathing. A colloidal oatmeal-based moisturizer was recommended for application to the entire body. Clobetasol 0.05% cream was prescribed for the neck, cubital fossae, and axilla. Fluticasone 0.05% cream was prescribed for facial lesions, to be applied twice daily for 10 days and then tapered over the following 10 days. Bilastine 20 mg daily, a nonsedative antihistamine, was also initiated.

For the maintenance therapy, the patient was prescribed pimecrolimus 1% cream to be applied for five days per week, topical corticosteroids (TCSs) to be applied for two days per week for six weeks, and continued use of a non-soap cleanser and colloidal oatmeal-based moisturizer.

The colloidal oatmeal-based moisturizer contributed to the improvement in the patient's condition from baseline to six weeks after treatment, as shown in Figure 1. The patient is currently in remission and has discontinued the use of TCSs. Maintenance therapy includes the application of pimecrolimus 1% cream twice weekly. Absence of itching, improved skin condition, better sleep, reduced discomfort, and improved social life were reported by the patient. The patient’s QoL notably improved, and the flare-up-free interval was six months.

**Figure 1:**
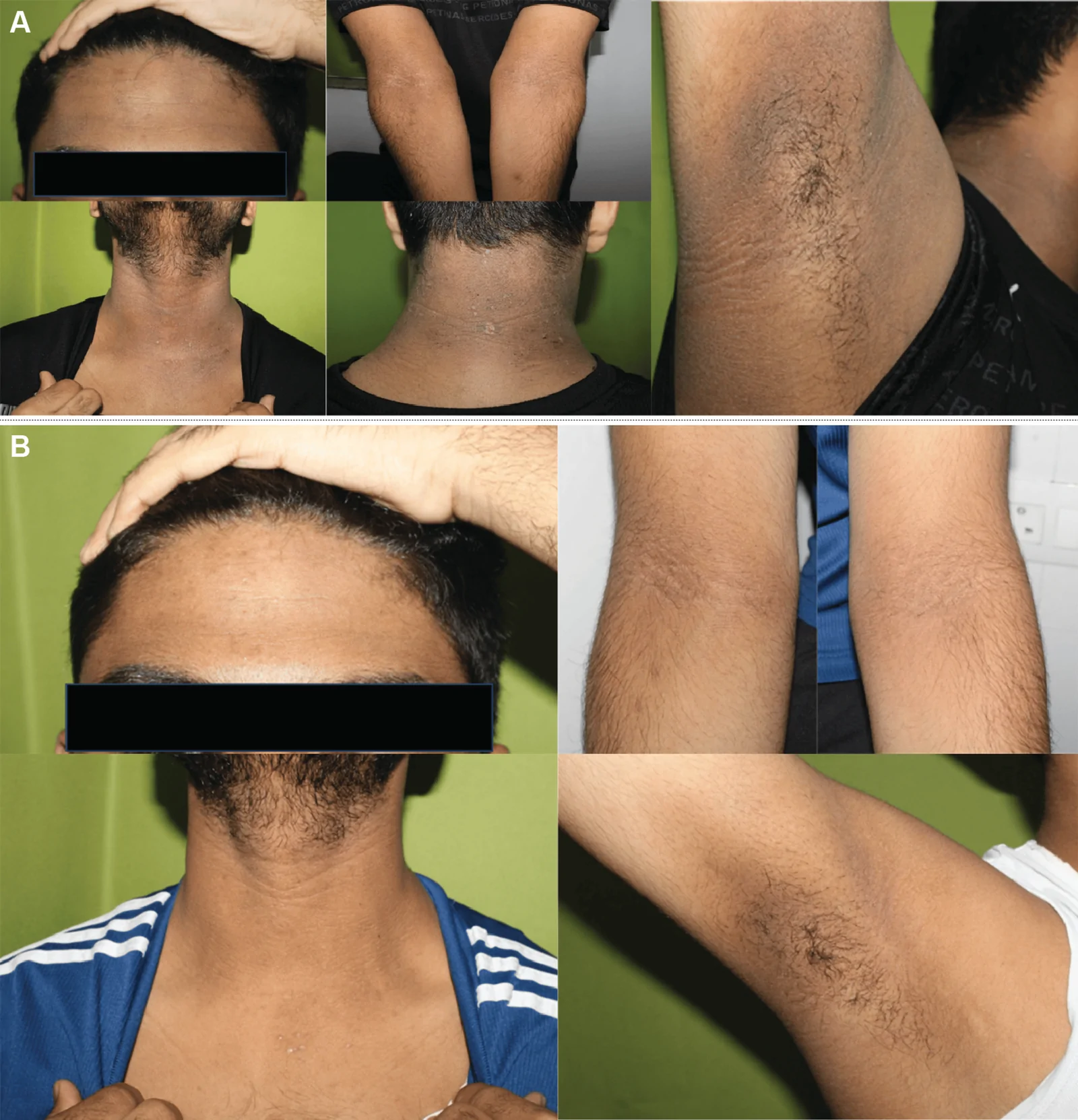
Affected body areas in Case 1 (AD): Forehead, elbow pits, anterior and posterior neck regions, and armpit A) Pretreatment; B) Post-treatment. AD: Atopic dermatitis.

Case 2 (severe eczema craquelé)

A 55-year-old man presented with severe skin dryness for two years, occasional itching, and worsening symptoms in the winter. The patient had a medical history of dyslipidemia and hypertension and was on atorvastatin and a telmisartan-thiazide combination. No family history of dyslipidemia, hypertension, or dermatological conditions was reported. On examination, skin dryness, severe itching, and eczematous lesions over the back were noted. A diagnosis of severe eczema craquelé was established. The condition developed approximately one year after the initiation of atorvastatin and telmisartan-thiazide therapy; therefore, medication-associated xerosis was considered a possible contributing factor.

The patient was prescribed oral eicosapentaenoic acid-docosahexaenoic acid capsules to be taken twice daily. Application of a colloidal oatmeal-based moisturizer was advised twice a day. For routine cleansing, the patient was instructed to use a syndet bar only while bathing, every other day.

An improvement in skin appearance was observed (Figure 2), with complete resolution of eczematous lesions and absence of itching achieved within 45 days of treatment with a colloidal oatmeal-based moisturizer. No side effects were reported during the treatment period.

**Figure 2:**
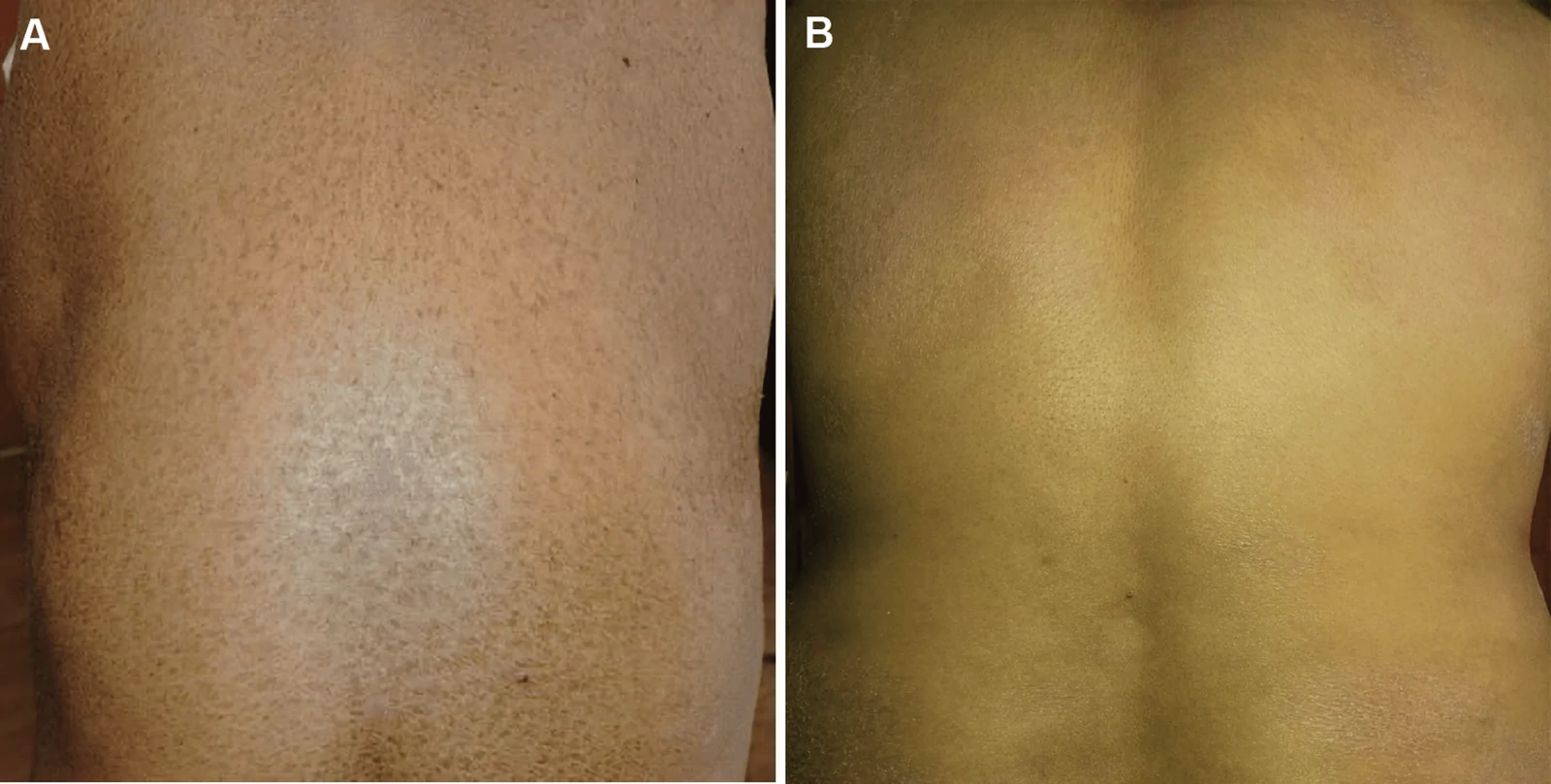
Skin appearance in Case 2 (severe eczema craquelé) A) Pretreatment; B) Post-treatment.

Case 3 (lichen amyloidosis with xerosis)

A 53-year-old man presented with itching, skin thickening, pigmentation, and excoriation marks. He was diagnosed with lichen amyloidosis associated with xerosis.

The patient was prescribed the regular use of colloidal oatmeal-based moisturizer to be applied twice daily to help restore skin barrier function and reduce itch. This was combined with keratolytic agents. The colloidal oatmeal-based moisturizer acted as supportive therapy, complementing the effects of the keratolytic treatment.

The colloidal oatmeal-based moisturizer, along with keratolytic support, improved the patient’s condition. Within 10-12 weeks, there was flattening of papules, improved skin hydration, and a noticeable reduction in hyperpigmented patches (Figure 3).

**Figure 3:**
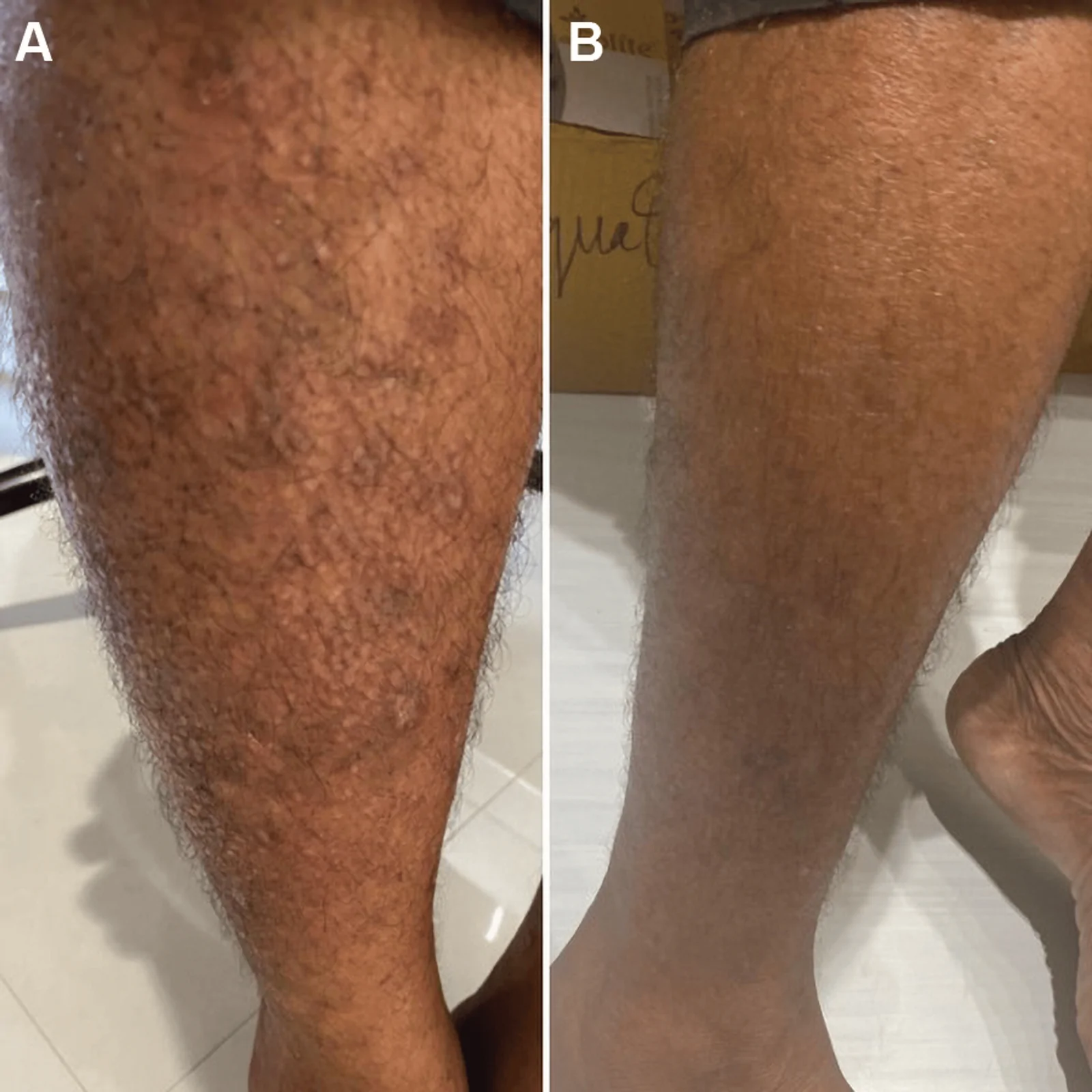
Affected lower limbs in Case 3 (lichen amyloidosis with xerosis) A) Pretreatment; B) Post-treatment.

Case 4 (AD)

A 14-year-old girl presented with itchy lesions over the face for the past few weeks. Medical history included intermittent use of topical steroids for skin lesions. Seasonal changes triggered flare-ups. There was no family history of AD.

On examination, diffuse erythema, scaling, and excoriation marks were observed over the face, particularly in the periorbital areas. The patient was diagnosed with AD. During the initial treatment, the patient was prescribed a colloidal oatmeal-based moisturizer for application to the entire body. A TCS, hydrocortisone, was prescribed for two weeks.

During the follow-up of six months, the use of the moisturizer was continued regularly. The TCS was discontinued after two weeks. The patient was advised to avoid known triggers. Improvement in itching was noted. After two weeks of treatment, there was a reduction in erythema and scaling (Figure 4). Treatment with colloidal oatmeal-based moisturizer as an adjuvant resulted in improvement in the patient’s QoL, reduced disease severity, and resulted in a flare-free interval of six months.

**Figure 4:**
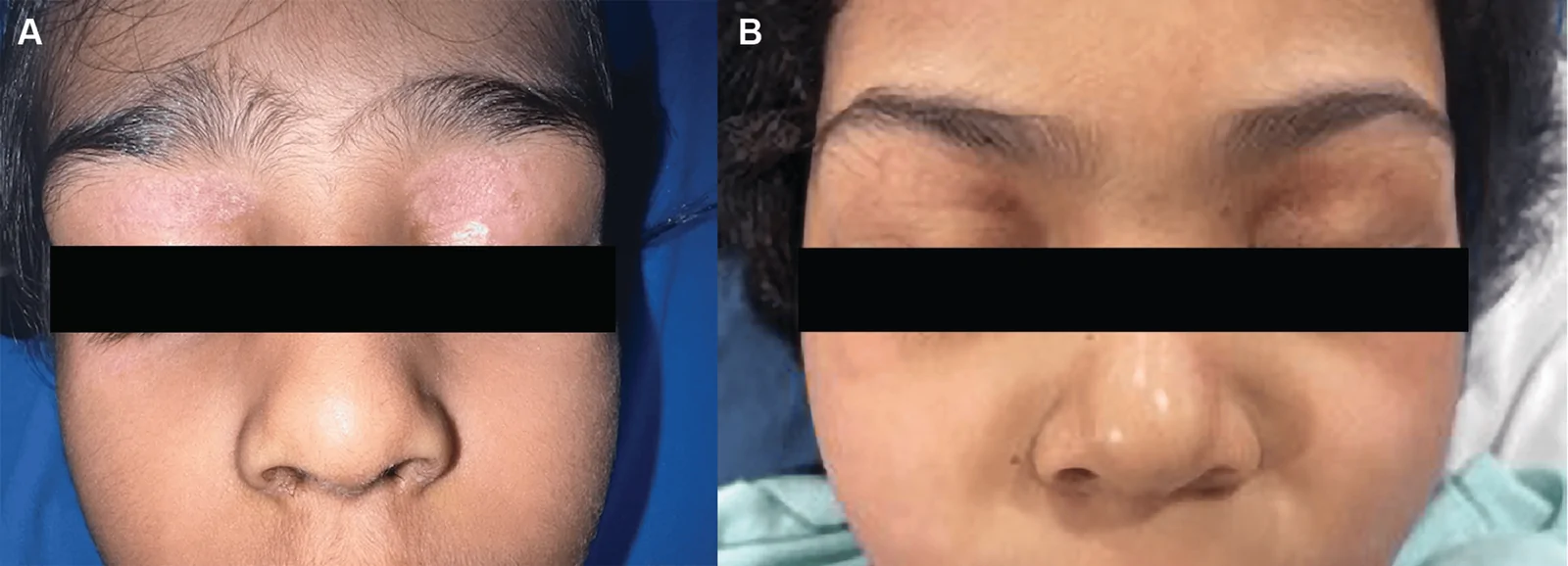
Affected facial periorbital area in Case 4 (AD) A) Pretreatment; B) Post-treatment. AD: Atopic dermatitis.

Case 5 (AD)

A 45-year-old woman presented with itchy lesions over the abdomen and limbs that had been present since early childhood. The patient reported recent flare-ups on both lower limbs. She had a history of intermittent use of topical steroids for skin lesions. Seasonal changes triggered the flare-ups. There was no family history of AD.

Diffuse erythema, scaling, and excoriation marks were observed over the trunk and bilateral lower limbs. Hyperpigmentation and lichenification were also noted all over the limbs. The patient was diagnosed with AD.

During the initial treatment, the patient was prescribed oral cyclosporine at a dose of 3 mg/kg for one month. A colloidal oatmeal-based moisturizer was recommended for application over the entire body. A TCS, mometasone, was prescribed for flare lesions.

During the follow-up of six weeks, regular use of the moisturizer was continued. The patient was advised to avoid known triggers. Improvement in itching and better sleep were noted. After six weeks, there was a reduction in erythema and scaling (Figure 5). The patient reported better sleep, increased comfort, and improved social life. Overall clinical improvement, including improvement in QoL and reduction in disease severity, was observed following treatment with oral cyclosporine, topical corticosteroids, and the colloidal oatmeal-based moisturizer. The patient experienced a flare-free interval of six months.

**Figure 5:**
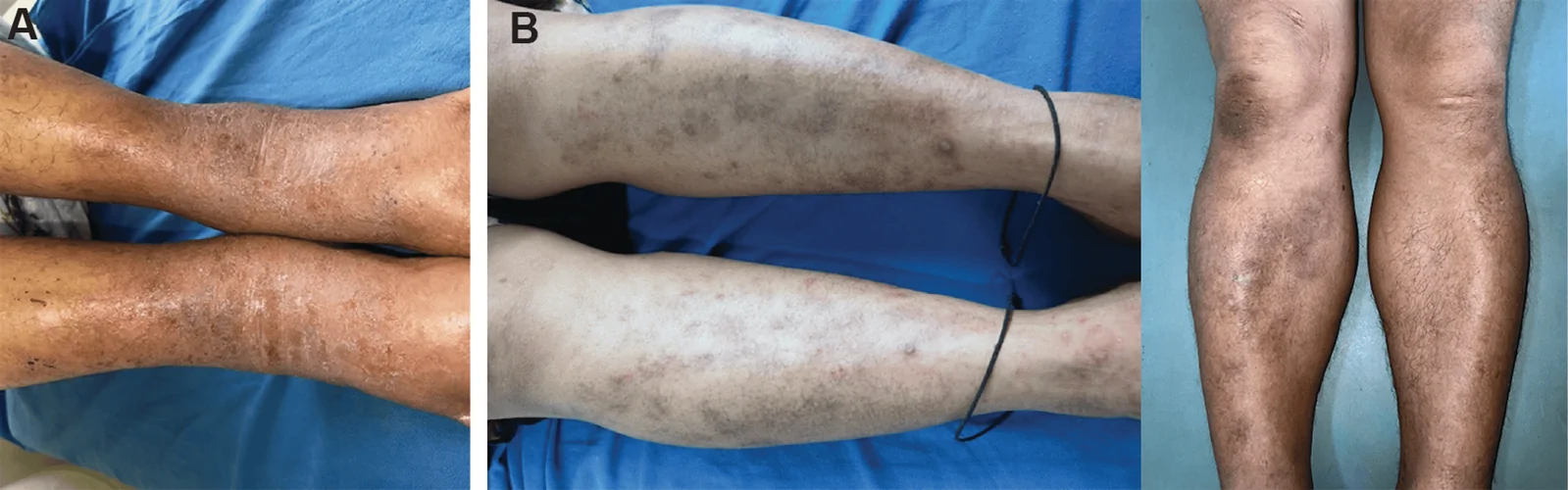
Affected lower limbs in Case 5 (AD) A) Pretreatment; B) Post-treatment. AD: Atopic dermatitis.

Case 6 (AD)

A female child aged 12 years presented with dry, scaly, and excoriated skin on the back of her thighs. The condition began two years ago and steadily worsened over time. There was no relevant family history, and no asthma was reported.

The patient exhibited chronic itching, excoriations, and dry skin, with periodic exacerbations. She was diagnosed with atopic eczema. Initial treatment included the application of mometasone cream once daily for two weeks, along with a colloidal oatmeal-based moisturizer applied twice daily. The maintenance therapy consisted of oral cetirizine and continued use of the colloidal oatmeal-based moisturizer twice daily.

Currently, the patient's condition has vastly improved with the regimen consisting of colloidal oatmeal-based moisturizer (Figure 6). Maintenance therapy with colloidal oatmeal-based moisturizer was continued. To date, the patient remains stable with ongoing good recovery.

**Figure 6:**
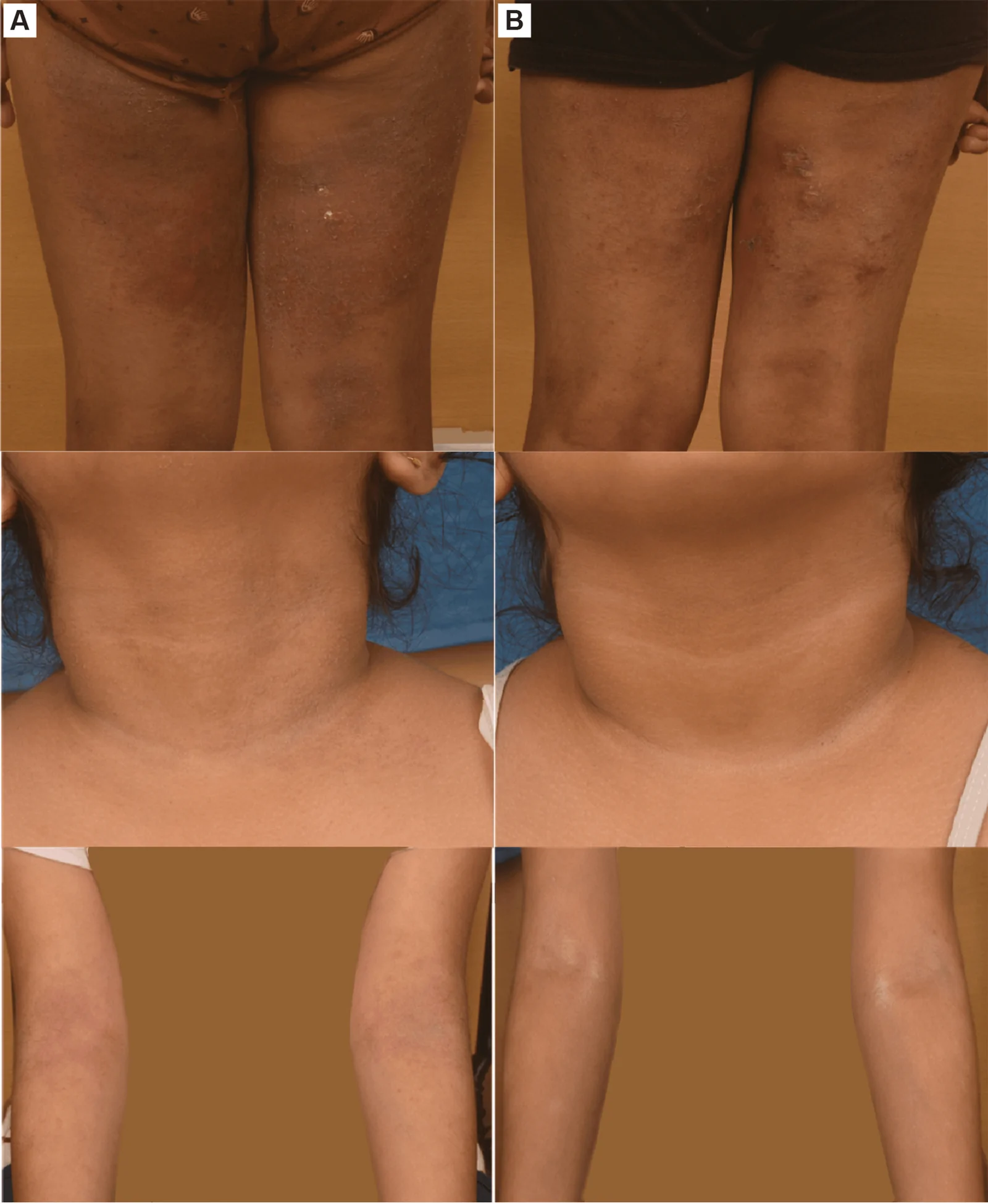
Affected body areas in Case 6 (AD): The back of the thighs, anterior neck, and arms A) Pretreatment; B) Post-treatment. AD: Atopic dermatitis.

## Discussion

This case series describes six patients with AD and associated dermatological conditions, such as severe eczema craquelé and lichen amyloidosis with xerosis, who were managed using a colloidal oatmeal-based moisturizer, either as a primary agent or as adjuvant therapy during the initial or maintenance phases of the treatment.

In the four cases with AD in the present series, the use of colloidal oatmeal-based moisturizer along with topical corticosteroids was associated with observed reductions in erythema, scaling, and disease severity, along with improvements in sleep, comfort, and overall QoL. As an adjuvant therapy, colloidal oatmeal prevented flare-ups during remission and supported both acute and maintenance phases. The patients also experienced flare-free intervals with continued moisturizer use, highlighting its role in both initial and maintenance therapy. However, these observations cannot be attributed specifically to the moisturizer alone given the concomitant treatments. Similar findings are reported in the literature wherein a review of multiple studies involving patients aged three months to 60 years with AD revealed that using moisturizers and/or cleansers containing colloidal oatmeal as adjunct therapy consistently improved disease severity, itch, skin dryness, and QoL [17].

Similarly, two clinical trials conducted in patients with mild to moderate AD (Study 1: age ≥8 years; Study 2: age ≥10 years) evaluated the efficacy of a 1% colloidal oatmeal cream applied for 14 days. In Study 1 (n=29; mean age: 27.1 years), improvements in AD severity and itch were observed following application of the 1% colloidal oatmeal cream and were maintained through Day 14. By Day 3, ≥20.0% improvement from baseline was observed in 53.6% of patients for AD severity, 25.0% for investigator-assessed AD severity, and 37.9% for itch; by Day 14, these proportions increased to 82.8%, 62.1%, and 85.7%, respectively. Study 2 (n=30; mean age: 32.9 years) also reported significant improvements in itch severity and AD severity, sustained through Day 14, with the application of colloidal oatmeal. Transepidermal water loss decreased significantly, while skin hydration increased at all time points [13]. These findings align with those of the AD cases in the present case series, where the use of colloidal oatmeal-based emollient improved skin condition, reduced disease severity, prevented flare-ups during the remission period, and ultimately enhanced QoL. However, differences in study design, treatment regimens, and outcome assessment preclude direct comparison.

Guidelines recommend the consistent use of emollients across all phases of AD treatment, including the maintenance phase [18,19]. Despite symptomatic relief, discontinuation of emollient use is not recommended, as regular and liberal application plays an important role in preserving the skin barrier, even in cases without visible lesions. In addition, long-term control of AD relies on preventing flare-ups through the avoidance of known triggers and adherence to routine skin care, particularly cleansing and moisturizing [20].

In the present series, a severe case of eczema craquelé induced by thiazide use was managed with colloidal oatmeal-based moisturizer alone as the primary treatment. In a clinical study, 1% colloidal oatmeal eczema cream with a non-fragranced standard moisturizer over 14 days significantly improved skin microbiome composition and restored barrier function in patients with mild to moderate eczema. Treatment resulted in a 54% reduction in the mean AD severity index, along with improvements in skin pH, barrier integrity, and hydration. The standard moisturizer increased hydration by only 36% at lesion sites compared with a 266% increase with the 1% colloidal oatmeal eczema cream [15]. These findings resonate with the case of severe eczema craquelé, which showed complete resolution of eczematous lesions; however, the contribution of the colloidal oatmeal-based moisturizer cannot be determined independently from the other aspects of management of the condition.

In another case involving lichen amyloidosis with xerosis, improvement in skin hydration and reduction in hyperpigmented patches were noted when a colloidal oatmeal-based moisturizer was used alongside keratolytic agents. Colloidal oatmeal is a commonly used skin protectant with a well-established role in managing xerotic skin conditions [20]. Colloidal oatmeal not only forms a protective film on the skin but also strengthens the water-binding and moisture-retention properties of the stratum corneum. In addition, colloidal oatmeal can also serve as a pH buffer, helping maintain skin surface pH [20]. A study evaluated four colloidal oatmeal extracts for anti-inflammatory and antioxidant properties and included an investigator-blind assessment in 29 women with dry, itchy skin on their lower legs to test a colloidal oatmeal skin protectant lotion. The extracts reduced proinflammatory cytokine levels in vitro, and the lotion demonstrated significant clinical improvements in skin dryness, scaling, roughness, and itch severity. These findings indicate that the direct antioxidant and anti-inflammatory properties of colloidal oatmeal contribute to its observed dermatological benefits in relieving dryness and itching [21].

In four cases, corticosteroids were prescribed during the initial treatment along with a colloidal oatmeal-based moisturizer. During the maintenance phase, corticosteroids were discontinued while the oatmeal-based moisturizer was continued, highlighting its potential role in maintaining disease control. This observation is further supported by a large-scale study involving both children and adults that analyzed data from 45,218 patients with and 9780 without colloidal oatmeal emollient prescriptions. After 1:1 matching (7846 patients per group), potent or very potent TCSs were prescribed to 42.45% of the non-emollient group versus 37.89% of the emollient group, with an odds of prescribing TCSs in the non-emollient group as 1.18 (95% confidence interval (CI): 1.10-1.26). Antimicrobial use was also higher in the non-emollient group (42.19% vs. 39.96%; odds ratio (OR) 1.13, 95% CI: 1.06-1.21) [22]. When prescribed as a first-line treatment, colloidal oatmeal emollient further reduced TCS and antimicrobial prescriptions. Only 10.24% of the emollient-first group received potent or very potent TCSs versus 16.14% in the subsequent-treatment group (OR 1.91, 95% CI: 1.49-2.46). Antimicrobial use was also lower in the emollient-first group (35.96% vs. 49.08%; OR 1.75, 95% CI: 1.50-2.04) [22]. These findings from the literature, together with observations from the present cases, highlight that early and consistent use of colloidal oatmeal-based emollients can support effective long-term management of AD; however, they do not establish a direct corticosteroid-sparing effect in the present case series.

The anti-inflammatory properties of colloidal oatmeal (especially avenanthramide) may provide a plausible biological basis for its potential supportive role in reducing the need for topical corticosteroids; however, this cannot be established from the present case series. Oatmeal extract reduces levels of arachidonic acid, cytosolic phospholipase A2, and tumor necrosis factor-alpha (TNF-α). It also inhibits nuclear factor kappa B (NF-κB) activity in keratinocytes and suppresses the release of proinflammatory cytokines and histamine, key drivers in the pathophysiology of inflammatory skin conditions. Notably, avenanthramides have been reported to block TNF-α-induced NF-κB luciferase activity and reduce proinflammatory cytokine IL-8 secretion [23]. These mechanisms provide a plausible biological basis for a potential supportive effect of colloidal oatmeal; however, they do not establish that these mechanisms were responsible for the clinical changes observed in the present cases. The observations from this series are consistent with prior studies demonstrating the anti-inflammatory, barrier-restoring, and skin health-promoting effects of colloidal oatmeal, while the present case series remains descriptive and hypothesis-generating.

Limitations

This case series has some limitations. The retrospective design and small sample size limit the generalizability of the findings and preclude meaningful assessment of the consistency of treatment responses across a broader patient population. The absence of a control or comparator group means that the observed clinical improvements cannot be definitively attributed to the colloidal oatmeal-based moisturizer alone, as spontaneous improvement, regression to the mean, the natural course of disease, or concurrent treatments may have contributed to the observed outcomes. In addition, treatment regimens varied across cases, with some patients receiving concomitant therapies such as topical corticosteroids, cyclosporine, antihistamines, or keratolytic agents, making it difficult to isolate the specific contribution of the emollient. The heterogeneity of the clinical presentations and treatment regimens further limits comparisons across cases. Follow-up duration was also variable, and the study relied primarily on descriptive clinical assessments, with standardized and validated measures of disease severity not consistently available across patients. The absence of standardized and validated outcome measures limits objective assessment and comparison of treatment response across cases. As a retrospective case series, the possibility of selection bias in case identification and reporting cannot be excluded. The relatively small number of cases and variable follow-up also limit conclusions regarding long-term treatment effects, relapse rates, and sustained tolerability. Safety was not assessed using a standardized or predefined framework; therefore, the absence of reported adverse events should not be interpreted as evidence of treatment safety.

## Conclusions

This case series describes clinical observations following the use of colloidal oatmeal-based moisturizer in the management of AD and associated dermatologic conditions like severe eczema craquelé and lichen amyloidosis with xerosis. Regular use of colloidal oatmeal-based moisturizer improved skin hydration, reduced erythema and scaling, enhanced patient comfort, and helped maintain remission, while corticosteroids were discontinued during the maintenance phase in some cases. These observations suggest a potential supportive role for colloidal oatmeal-based moisturizer as part of both acute and maintenance phases of comprehensive management; however, the findings do not establish treatment efficacy or a corticosteroid-sparing effect. Further prospective studies using standardized outcome measures are warranted to evaluate its clinical effects.
